# *Porphyridium cruentum*–enriched *Artemia* enhances survival, growth, and viral resilience of glass eels (*Anguilla bicolor bicolor*) under endemic *Anguillid* *herpesvirus*-1 conditions

**DOI:** 10.14202/vetworld.2026.1402-1416

**Published:** 2026-04-12

**Authors:** Adang Saputra, Lusi H. Suryaningrum, Endhay K. M. Kontara, Abidin Nur, Edy B. Kholidin, Taukhid Taukhid, Yohanna R. Widyastuti, Reza Samsudin, Siti Murniasih, Lisa Ruliaty, Tri H. Prihadi, Brata Pantjara, Tatag Budiardi, Maya Meiyana, Eri Setiadi, Haryono Haryono

**Affiliations:** 1 Research Center for Freshwater Aquaculture, National Research and Innovation Agency (BRIN), Cibinong, Indonesia; 2 Research Center for Applied Zoology, National Research and Innovation Agency (BRIN), Cibinong, Indonesia; 3 Department of Aquaculture, Faculty of Fisheries and Marine Science, Bogor Agricultural University (IPB University), Bogor 16680, Indonesia; 4 Research Center for Biosystematics and Evolution, National Research and Innovation Agency (BRIN), Cibinong, Indonesia

**Keywords:** *Anguilla bicolor bicolor*, *Anguillid herpesvirus-1*, *Artemia* enrichment, eel aquaculture, glass eel survival, *Porphyridium cruentum*, tropical eel culture, viral resilience

## Abstract

**Background and Aim:**

The tropical eel *Anguilla bicolor bicolor* is a valuable aquaculture commodity; however, the survival of glass eels during the early nursery stage remains low due to nutritional limitations and vulnerability to viral infections, especially *Anguillid herpesvirus-1* (AngHV-1). Using microalgae to enrich live feed has been suggested as a strategy to enhance larval nutrition and resilience in endemic disease conditions. This study examined the effects of *Artemia* enriched with the red microalgae *Porphyridium cruentum* on the nutritional quality of live feed, survival and growth of glass eels, intestinal structure, water quality, bacterial dynamics, and AngHV-1 infection status under hatchery-like conditions.

**Materials and Methods:**

A completely randomized design with seven treatments was employed: an unenriched control and six enrichment combinations based on *P. cruentum* cell density and enrichment duration (3 × 10^5^ cells/mL for 6 h; 3 × 10^5^ cells/mL for 12 h; 6 × 10^5^ cells/mL for 6 h; 6 × 10^5^ cells/mL for 12 h; 9 × 10^5^ cells/mL for 6 h; and 9 × 10^5^ cells/mL for 12 h), each with three replicates. Glass eels (0.12 ± 0.01 g; 4.96 ± 0.09 cm) were stocked at 90 fish per tank in 70-L containers and reared for 60 days, fed exclusively with either enriched or unenriched *Artemia*. Evaluations included the proximate composition of enriched *Artemia*, survival rate, growth performance, intestinal villus morphometry, water quality parameters, total heterotrophic bacterial counts, and the presence of AngHV-1 verified through conventional polymerase chain reaction.

**Results:**

Enrichment substantially enhanced the nutritional profile of *Artemia*, increasing crude protein to 61.48% and lipid content to 9.98% on a dry matter basis. The treatment with *P. cruentum* at 6 × 10^5^ cells/mL for 6 h yielded the highest survival rate (60.74 ± 3.9%) and specific growth rate (1.11 ± 0.1%/day), both significantly higher than those in the control and other treatments (p < 0.05). Intestinal villus length and surface area showed no significant differences among treatments (p > 0.05). Water quality remained within suitable ranges for glass eel culture, and bacterial counts, although slightly higher in enriched treatments, stayed below harmful thresholds. AngHV-1 was detected in all groups, but the enriched treatment at 6 × 10^5^ cells/mL for 6 h exhibited the faintest viral DNA band, indicating a reduction in apparent viral signal.

**Conclusion:**

Short-term enrichment of *Artemia* with *P. cruentum*, especially at 6 × 10^5^ cells/mL for 6 h, notably improves the survival and growth performance of *A. bicolor bicolor* glass eels without harming intestinal morphology or water quality. The lower AngHV-1 polymerase chain reaction signal indicates a possible role of nutritionally enriched live feed in boosting host resilience and reducing viral activity under endemic infection conditions. This enrichment method offers a practical and non-drug approach to enhance early-stage eel aquaculture performance.

## INTRODUCTION

Tropical eel aquaculture encounters a major hurdle at the glass eel stage, where high and unpredictable mortality rates severely hinder production efficiency and industry growth [1–4]. In Indonesia and other producing countries, *A. bicolor bicolor* glass eels are still entirely collected from the wild because reliable artificial breeding methods have not yet been developed [5, 6]. Wild-caught glass eels often carry viral and bacterial pathogens acquired during their marine and estuarine life stages, making them highly susceptible to disease outbreaks when transferred to intensive farming systems [1–3]. Among these pathogens, *Anguillid herpesvirus-1* (AngHV-1) has been repeatedly identified as a primary cause of mortality in both European and tropical eel species, with high prevalence in wild and farmed populations [1–4]. The reliance on infected wild seed and the lack of effective antiviral treatments make AngHV-1 a significant obstacle to sustainable eel aquaculture [1–4].

Herpesviruses establish lifelong latency in their hosts and can reactivate under stress or immunosuppression, leading to recurrent disease episodes rather than complete clearance [1, 3]. In the case of AngHV-1, eradication from wild glass eel stocks is not feasible with current tools, and direct antiviral drugs are not available for aquaculture use [1–4]. Consequently, disease management in eel hatcheries and nurseries must rely on non-pharmacological strategies that enhance host resilience, limit viral replication, and keep infections in a subclinical or latent state [1, 3]. Previous studies have mainly focused on pathogen detection, epidemiology, or outbreak description, with relatively few efforts to explore nutritional or functional feed approaches as tools to modulate AngHV-1 infection dynamics under endemic conditions [1–4, 7].

Adequate nutrition is essential for the survival and growth of glass eels during the transition from yolk dependence to external feeding [8, 9]. In early life stages, fish larvae and glass eels have high metabolic rates but limited digestive capacity; therefore, they rely on live feeds with suitable particle size, digestibility, and nutrient content [5, 8–10]. *Artemia nauplii* are still the most commonly used live feed in marine and brackishwater fish culture, including eel nurseries, but their natural levels of long-chain polyunsaturated fatty acids (LC-PUFA), vitamins, and antioxidants are often insufficient [5, 6, 10]. To address these issues, enriching *Artemia* with microalgae or commercial emulsions has become standard practice, especially to boost n-3 highly unsaturated fatty acids (HUFA), such as eicosapentaenoic acid (EPA) and docosahexaenoic acid (DHA), which are vital for growth, neural development, and stress resistance in fish larvae [5, 6, 10, 11]. However, most research on *Artemia* enrichment has concentrated on marine finfish and crustaceans, focusing on growth and survival in pathogen-free settings, and rarely considering viral disease scenarios or glass eels [5, 6, 10, 11].

The red microalgae *Porphyridium cruentum* is increasingly seen as a promising functional ingredient for aquaculture because of its unique biochemical profile [8, 11]. Besides its relatively high protein and lipid contents, *P. cruentum* produces large amounts of PUFA (including EPA), phycobiliproteins (such as phycoerythrin), carotenoids, and sulfated polysaccharides with antioxidant, immunomodulatory, and antiviral properties in various aquatic and mammalian models [8, 11]. These qualities indicate that *P. cruentum* could serve not only as a nutritional supplement for live feeds but also as a functional agent that can influence host–pathogen interactions and boost resistance against viral infections [8, 11]. However, the use of *P. cruentum* in live-feed enrichment for eel culture is still largely unexplored, and its potential to influence AngHV-1 infection has not been proven through experiments [6, 11].

Furthermore, most studies on eel viral diseases have used challenge models or focused on relatively pathogen-free populations, while commercial hatcheries and nurseries usually face endemic infection pressure, with glass eels already AngHV-1 positive at stocking [1–3]. This discrepancy raises questions about the relevance of traditional challenge trials to real industry conditions [1, 3]. Therefore, there is a need for experimental approaches that simulate actual hatchery scenarios, where the goal is not to prevent initial infection but to suppress viral replication and lessen clinical impacts in already infected glass eel groups [1, 3]. In this context, nutritional strategies that combine better growth performance with partial viral suppression, without relying on drugs or vaccines, could be a crucial step toward creating more biosecure and sustainable fish aquaculture systems [1, 3, 9].

Despite the growing recognition of viral diseases as major challenges in eel aquaculture, most studies on AngHV-1 have mainly concentrated on virus detection, epidemiology, and pathological features rather than on practical mitigation strategies suitable for hatcheries. In particular, the early nursery stage of *A. bicolor bicolor* remains poorly studied from a nutritional disease-management perspective, even though this stage is linked to the highest mortality rates in commercial production systems. Previous research on *Artemia* enrichment has mostly targeted marine finfish and crustacean larvae in pathogen-free experimental conditions, primarily aiming to improve growth and survival by enhancing essential fatty acids such as EPA and DHA. However, there is very limited information on how enriched live feeds affect host resilience against viral infections in eel culture, especially under endemic infection scenarios where glass eels already carry AngHV-1 at stocking. Additionally, although *P. cruentum* is known to contain bioactive compounds like PUFA, phycobiliproteins, carotenoids, and sulfated polysaccharides with antioxidant and immunomodulatory properties, its use as a functional enrichment agent for live feed in eel aquaculture has received little scientific attention. To date, no comprehensive study has simultaneously assessed the nutritional enhancement of *Artemia*, the growth and survival of glass eels, intestinal histological responses, microbial dynamics, water quality stability, and AngHV-1 infection status under hatchery-like conditions. This significant lack of integrated data leaves an important knowledge gap, especially for hatcheries relying on wild-caught glass eels already exposed to viral pathogens.

Therefore, the current study aimed to assess the effectiveness of *Artemia* enriched with *P. cruentum* as a functional live feed for enhancing the performance of *A. bicolor bicolor* glass eels under conditions where AngHV-1 is endemic. Specifically, this research examined how different combinations of *P. cruentum* cell density and enrichment duration affected several key biological and environmental factors relevant to early eel cultivation. These included: (i) the proximate composition and estimated energy value of enriched *Artemia*; (ii) the survival rate and growth performance of glass eels during the nursery stage; (iii) intestinal villus morphology as an indicator of digestive capacity; (iv) water quality parameters and bacterial activity in the rearing environment; and (v) the detection and relative severity of AngHV-1 infection via polymerase chain reaction. The results are expected to offer practical insights for developing nutritionally based management strategies that support sustainable tropical eel aquaculture production.

## MATERIALS AND METHODS

### Ethical approval

All experimental procedures involving *Anguilla bicolor bicolor* were conducted in accordance with the institutional guidelines for the care and use of aquatic animals in research and were reviewed and approved by the Ethical Committee of the National Research and Innovation Agency (BRIN), Indonesia, under approval number 253/KE.02/SK/11/2024. The approved protocol covered all stages of the study, including the collection, transportation, acclimatization, stocking, rearing, feeding, handling, sampling, and euthanasia of glass eels used in the experiment.

Particular attention was given to minimizing animal stress and unnecessary suffering throughout the study. Glass eels collected from the Cimandiri River estuary were transported in oxygenated double-layer plastic bags placed in insulated containers with ice gel packs to maintain suitable conditions during transit. On arrival at the experimental facility, the fish were acclimatized for 7 days before the feeding trial to allow recovery from transport-related stress. During the 60-day rearing period, fish were maintained under controlled environmental conditions with continuous aeration and water quality kept within acceptable ranges for eel culture. Handling during routine management and sampling was performed carefully and only when necessary to reduce physical disturbance and stress.

At the end of the experiment, fish sampling for growth assessment, histological examination, and molecular detection of AngHV-1 was carried out in accordance with the approved ethical protocol. The number of fish sampled was kept to the minimum required to achieve the scientific objectives while ensuring reliable data collection. Thus, the study complied with recognized animal welfare principles for the humane use of aquatic organisms in experimental research.

### Study period and location

The experiment was conducted over a 5-month period from December 2024 to April 2025 at the Brackishwater Aquaculture Development Center (BADC), Jepara Subdistrict, Jepara Regency, Central Java Province, Indonesia (59418).

### Source of *P. cruentum* and glass eels

The inoculum of *P. cruentum* was obtained from pure stock cultures maintained at BADC, Jepara, during the intermediate (exponential) growth phase in 20 L glass carboys to ensure culture purity and sufficient cell density. Cultures were kept in f/2 medium [12] at 30 g/L (approximately 30 ppt) salinity and 24°C ± 1°C, under a 12 h light: 12 h dark photoperiod with continuous aeration.

Glass eels of *A. bicolor bicolor* were collected from local fishers operating in the Cimandiri River estuary, Pelabuhan Ratu Subdistrict, Sukabumi Regency, West Java Province, Indonesia. For transportation, the glass eels were packed in double-layer plastic bags containing 3 L of water with a biomass density of approximately 200 g per bag. The bags were oxygenated and placed in insulated foam boxes containing four ice gel packs (500 g; 20 × 15 × 3 cm). No water exchange was conducted during transport. Upon arrival at the experimental facility, the glass eels were acclimatized for 7 days in 0.5 m³ fiberglass tanks to recover from transport stress and were fed *Artemia* ad libitum.

### Experimental tanks and rearing conditions

After acclimation, glass eels were randomly placed into 70 L plastic containers (53.5 × 37.5 × 32.0 cm³) with a water volume of 30 L. The containers were situated in a semi-indoor facility (200 m²) under a 12 h light and 12 h dark photoperiod. Each container had a single aeration point connected to a root blower system.

Water used for the culture system was sourced from a borewell and allowed to settle for 3 days in a 10 m³ aerated concrete tank. The water was then supplied to the experimental tanks using a 30 W submersible pump through dual cartridge filtration units (Kinnoyama, Nanotech @ SG Clear). The initial mean body weight and total length of the glass eels were 0.12 ± 0.01 g and 4.96 ± 0.09 cm, respectively. Fish were stocked at a density of 90 individuals per tank and reared for 60 days under the respective feeding treatments.

### Hatching and enrichment of *Artemia* with *P. cruentum*

*Artemia* cysts (Supreme Plus, Golden West®, Great Salt Lake, USA) were used as live feed. Cysts were hatched following standard procedures commonly used for commercial *Artemia* production in marine fish larviculture. Briefly, cysts were incubated in conical hatching tanks containing artificial seawater at 30 g/L salinity and 28°C ± 1°C with continuous aeration and illumination for 24 h to obtain instar-I nauplii [13, 14].

For enrichment, newly hatched nauplii were stocked at approximately 300–500 individuals/L in 5 L cylindrical-conical vessels containing seawater at 30 g/L salinity and 28°C ± 1°C. Strong aeration was provided to maintain suspension of both *Artemia* and microalgal cells. The experimental design consisted of one control treatment (unenriched *Artemia*) and six enrichment treatments with different combinations of *P. cruentum* cell density and enrichment duration: control (no enrichment), A (3 × 10^5^ cells/mL for 6 h), B (3 × 10^5^ cells/mL for 12 h), C (6 × 10^5^ cells/mL for 6 h), D (6 × 10^5^ cells/mL for 12 h), E (9 × 10^5^ cells/mL for 6 h), and F (9 × 10^5^ cells/mL for 12 h). Each treatment was replicated three times (n = 3 tanks). Throughout the experimental period, glass eels were fed exclusively with *Artemia* corresponding to the assigned enrichment treatment.

### Growth performance and parameter measurements

At the start of the experiment, 50 fish were randomly sampled to determine initial body weight and total length. At the end of the 60-day trial, all fish from each tank were individually measured to assess final body weight and total length. The growth parameters evaluated included survival rate (SR) [16], average weight gain (AWG) [17], and specific growth rate (SGR).

SR (%) was calculated as: SR = 100 × (Nf/Ni), where Nf represents the final number of fish and Ni represents the initial number of fish.

AWG (g) was calculated as: AWG = Wf − Wi, where Wf and Wi represent the final and initial mean body weights, respectively.

SGR (%/day) was calculated as: SGR = 100 × (ln Wf − ln Wi)/t, where t represents the rearing period (days).

### Proximate composition analysis of *Artemia*

The proximate composition of *Artemia* (moisture, crude protein, crude lipid, and ash) after enrichment with *P. cruentum* was determined using standard analytical methods [18–20]. For each treatment, three independent *Artemia* samples of approximately 30 g wet weight were collected at the end of the enrichment period. The samples were rinsed with distilled water, blotted dry, frozen at −20°C, freeze-dried, and homogenized using a mortar and pestle before analysis [21].

Moisture content was determined according to SNI 01-2891-1992. Crude protein was analyzed using the 18-8-31/MU titrimetric method, whereas crude lipid content was determined using method 18-8-5/MU (Weibull gravimetric method). Ash content was measured following SNI 01-2891-1992 section 6.1. Nitrogen-free extract (NFE, %) was calculated as: NFE = 100 − (moisture + protein + lipid + ash) [22]. Gross energy (kcal/100 g) was estimated using physiological fuel values of 4.0 kcal/g for protein and NFE and 9.0 kcal/g for lipid according to Atwater conversion factors [23, 24].

### Intestinal villi morphometric analysis

At the end of the experiment, one glass eel from each replicate tank was sampled for histological examination. Tissue samples were fixed in Bouin’s solution for 48 h and then dehydrated through graded ethanol concentrations (70%, 80%, 90%, 95%, and 100%), followed by immersion in an ethanol–xylol solution (1:1). The tissues were embedded in paraffin blocks and sectioned transversely at the abdominal region (from the posterior part of the stomach to the anus) using a microtome. Histological sections were stained with hematoxylin–eosin.

Microscopic observations were performed using an Olympus BX53 microscope (Japan) at 10× magnification. Villus length and surface area were calculated with the modified formula: villus surface area (µm²) = [(b + c)/2] × a, where a represents villus height or length, b represents apical villus width, and c represents basal villus width.

### Detection of AngHV-1 infection

Detection of AngHV-1 infection in the *A. bicolor bicolor* glass eel population was carried out twice: once before the experiment began and again at the end of the rearing period. Viral genome detection was performed qualitatively (semi-quantitatively) using conventional polymerase chain reaction based on the method described previously [25], with minor modifications.

For each treatment and sampling time, gill and kidney tissues from five glass eels (one fish from each replicate tank) were pooled to create a single DNA sample. Genomic DNA was extracted using a commercial tissue DNA extraction kit (Geneaid™ DNA Isolation Kit Ver. 06.22.16) following the manufacturer’s instructions. The quality and concentration of the extracted DNA were measured with a NanoDrop 2000 UV-Vis spectrophotometer (Thermo Scientific, WA, USA).

Detection of AngHV-1 was carried out using the primer pair HVAPOLVPSD and HVAPOLOOSN (Table 1), which amplify a 394-bp fragment of the viral genome [25]. PCR amplification was performed under the following conditions: initial denaturation at 94°C for 5 min (1 cycle), followed by 40 cycles of denaturation at 94°C for 30 s, primer annealing at 65°C for 45 s, and extension at 72°C for 60 s, with a final extension step at 72°C for 7 min. PCR products were stored at 4°C until analysis and subsequently visualized using 2% agarose gel electrophoresis to confirm the presence of viral DNA bands.

**Table 1:** Primers used for detection of *Anguillid herpesvirus 1.*

Primary name	Primary sequence	Reference
HVAPOLVPSD	5′-GTG TCG GGC TTT GTG GTG C-3′	[25]
HVAPOLOOSN	5′-CAT GCC GGG AGT CTT TTT GAT-3′	[25]

### Statistical analysis

All results are presented as mean ± standard error (SE), with the tank considered as the experimental unit. Before conducting statistical analysis, the data were tested for normality using the Shapiro–Wilk test and for homogeneity of variance with Levene’s test. When these assumptions were met, a one-way analysis of variance was used to assess the effect of treatments. If significant differences were found (p < 0.05), Duncan’s multiple range test was applied as a post hoc test to compare treatment means. Statistical analyses were conducted using SPSS software version 31 (IBM Corp., NY, USA).

## RESULTS AND DISCUSSION

### Nutrient composition of enriched *Artemia*

The proximate composition and energy content of enriched *Artemia* are shown in Table 2. Moisture content, expressed on a wet weight basis, ranged from 91.83% to 94.21%, while crude protein, crude lipid, ash, NFE, and gross energy were expressed on a dry matter basis. Compared to the control, enrichment consistently enhanced the nutritional value of *Artemia*. Crude protein increased from 47.72% in the control to 48.28%–61.48% in the enriched treatments, with the highest level observed in treatment A. Even greater increases were seen in crude lipid, which rose from <0.30% in the control to 4.72%–9.98% in the enriched groups, reaching a maximum in treatment F. This rise in lipid content contributed to higher fat-based energy, which increased from a negligible amount in the control to 3.29–5.67 kcal/100 g in the enriched treatments.

**Table 2:** Proximate composition of *Artemia nauplii*: moisture (% wet weight), crude protein, crude lipid, ash, nitrogen-free extract (NFE) (% dry matter), and gross energy (kcal/100 g dry matter) in control and *Porphyridium cruentum*-enriched treatments (A–F).

Parameter	Control	A	B	C	D	E	F
Moisture (%)	93.42	93.60	94.04	92.27	94.21	91.83	93.69
Crude protein (%)	47.72	61.48	48.28	52.59	49.14	51.68	51.26
Crude lipid content (%)	<0.30	6.33	7.88	4.72	6.21	5.69	9.98
Ash (%)	28.19	29.06	27.66	19.54	32.59	19.08	21.38
NFE* (%)	23.80	3.13	16.18	23.15	12.06	23.55	17.38
Total energy (kcal/100 g)	18.90	20.10	19.59	26.74	17.31	26.71	23.01

Total gross energy increased across all enriched groups, with the highest values seen in treatments C (26.74 kcal/100 g) and E (26.71 kcal/100 g), compared to 18.90 kcal/100 g in the control. Conversely, ash and NFE showed variable patterns among treatments. These findings suggest that enrichment with *P. cruentum* enhanced not only the macronutrient profile of *Artemia* but also its overall energy value.

The significant increase in crude protein and crude lipid highlights *P. cruentum*’s effectiveness as a bioencapsulation agent. *Artemia*’s active filtration behavior helps it absorb biomolecules from *P. cruentum*, including proteins, unsaturated fatty acids, and lipophilic pigments [26]. The biochemical makeup of *P. cruentum* includes protein (30%–43%), lipid (9%–14%), and EPA, which makes up about 9.7% of total fatty acids [27]. Additionally, compounds like sulfated polysaccharides and phycoerythrin further boost the nutritional and biological value of live feed [28, 29]. Therefore, enrichment with *P. cruentum* enhances both the quantity and quality of *Artemia*.

Among the tested treatments, C offered the most balanced nutrient profile, with crude protein at 52.59%, crude lipid at 4.72%, and total energy of 26.74 kcal/100 g. The 6 h enrichment period likely coincided with the peak ingestion phase of *Artemia* on microalgal cells [26], making it an efficient enrichment window [30]. The increased energy from lipid and pigment fractions, along with the antioxidant and bioactive properties of *P. cruentum*, may also enhance the immunoprotective value of enriched *Artemia* [27, 31]. Overall, these findings suggest that enrichment with *P. cruentum* improves *Artemia*’s nutritional profile and enhances its suitability as a strategic live feed for the early rearing phase of *A. bicolor bicolor*.

### Survival rate

**Figure 1:**
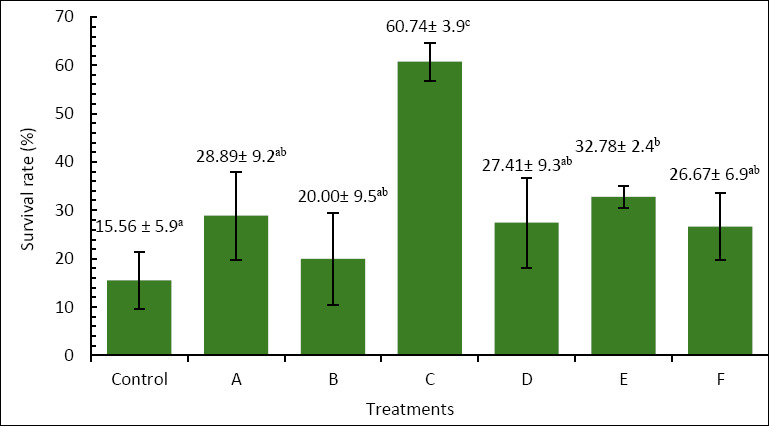
Survival (%) of *Anguilla bicolor bicolor* glass eels after 60 days of rearing under different combinations of *Artemia* enrichment density and duration using *Porphyridium cruentum*. Remark: Values followed by the same superscript letter are not significantly different (p > 0.05).

The survival of *A. bicolor bicolor* over the 60-day rearing period varied significantly among treatments (Figure 1). The control group had the lowest survival rate at 15.56% ± 5.9%, while treatment C achieved the highest survival at 60.74% ± 3.9%, which was significantly higher than all other groups (p < 0.05). Treatments A (28.89% ± 9.2%), B (20.00% ± 9.5%), D (27.41% ± 9.3%), and F (26.67% ± 6.9%) did not differ significantly from the control (*p* > 0.05). Treatment E (32.78% ± 2.4%) showed significantly higher survival than the control but remained lower than treatment C. These findings demonstrate that enriching *Artemia* with *P. cruentum* at 6 × 10^5^ cells/mL for 6 h was the most effective condition for improving survival during rearing.

The improved survival rate is likely due to the increased supply of nutrients and bioactive compounds transferred from *P. cruentum* to *Artemia*. Higher lipid and protein levels provide vital energy and substrates for larval development [32, 33]. Lipids act as a key energy reserve during metamorphosis, while proteins support tissue repair and growth. Additionally, PUFA, especially DHA, along with phycoerythrin from *P. cruentum*, may enhance energy metabolism, enzymatic activity, membrane fluidity, and osmoregulation [33, 34]. Functional compounds such as sulfated polysaccharides and EPA may also boost larval immunity and thus improve survival [28, 35].

### Growth performance

**Figure 2:**
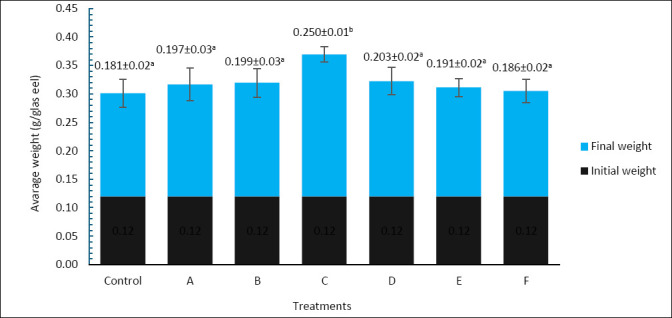
Final body weight (g) of *Anguilla bicolor bicolor* glass eels after 60 days of rearing under different combinations of *Artemia* enrichment density and duration using *Porphyridium cruentum*. Values followed by the same superscript letter are not significantly different (p > 0.05).

Final body weight after 60 days also varied significantly among treatments (Figure 2). The control group had the lowest final body weight, at 0.181 ± 0.02 g, while treatment C showed the highest value, at 0.250 ± 0.01 g, which was significantly higher than all other treatments (p < 0.05). Treatments A (0.197 ± 0.03 g), B (0.199 ± 0.03 g), D (0.203 ± 0.02 g), E (0.191 ± 0.02 g), and F (0.186 ± 0.02 g) did not differ significantly from the control (p > 0.05). These results suggest that treatment C was also the most effective in increasing final body weight.

**Figure 3:**
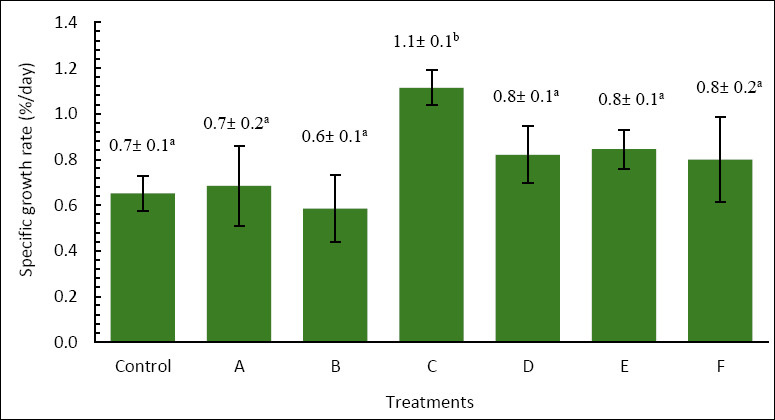
Specific growth rate (%/day) of *Anguilla bicolor bicolor* glass eels after 60 days of rearing under different combinations of *Artemia* enrichment density and duration using *Porphyridium cruentum*. Values followed by the same superscript letter are not significantly different (p > 0.05).

A similar pattern was observed for SGR (Figure 3). Treatment C yielded the highest SGR, at 1.11 ± 0.1%/day, which was significantly greater than the control (0.65 ± 0.1%/day) and all other treatments (p < 0.05). In contrast, treatments A (0.69 ± 0.2%/day), B (0.59 ± 0.6%/day), D (0.82 ± 0.1%/day), E (0.84 ± 0.1%/day), and F (0.80 ± 0.2%/day) did not differ significantly from the control (p > 0.05). Therefore, enriching *Artemia* with *P. cruentum* at 6 × 10^5^ cells/mL for 6 h produced the greatest improvement in both final body weight and SGR of *A. bicolor bicolor*.

These growth responses align with the nutrient profile shown in Table 2. Treatment C offered a balanced mix of crude protein, crude lipid, and total energy, ensuring an adequate supply of amino acids and energy for larval growth. Protein serves as the main substrate for tissue synthesis and enzyme production, while lipid, especially PUFA, functions as a key energy reserve during metamorphosis and developmental transitions [32, 33]. Since zooplankton naturally provide a major lipid source for larvae, enriching *Artemia* with *P. cruentum* may better mimic natural feeding conditions by increasing the availability of long-chain fatty acids essential for larval nutrition [33, 34].

The physiological value of *P. cruentum* is further supported by its high content of PUFA, including EPA and DHA precursors, as well as phycoerythrin and sulfated polysaccharides. These compounds may boost membrane fluidity, metabolic efficiency, osmoregulation, and immune responses [28, 34, 35]. Transferring these compounds into *Artemia* enhances the functional quality of the live feed by providing not just energy but also biologically active substances that aid larval physiology and resilience [36–38]. The simultaneous increase in survival and growth suggests that nutritional improvements benefited both parameters at the same time. Since these gains were not linked to changes in intestinal villus morphology, the improved performance in treatment C seems to be mainly due to better nutrient availability and bioactive quality rather than structural modifications of the digestive tract.

### Intestinal villi morphometric analysis

**Figure 4:**
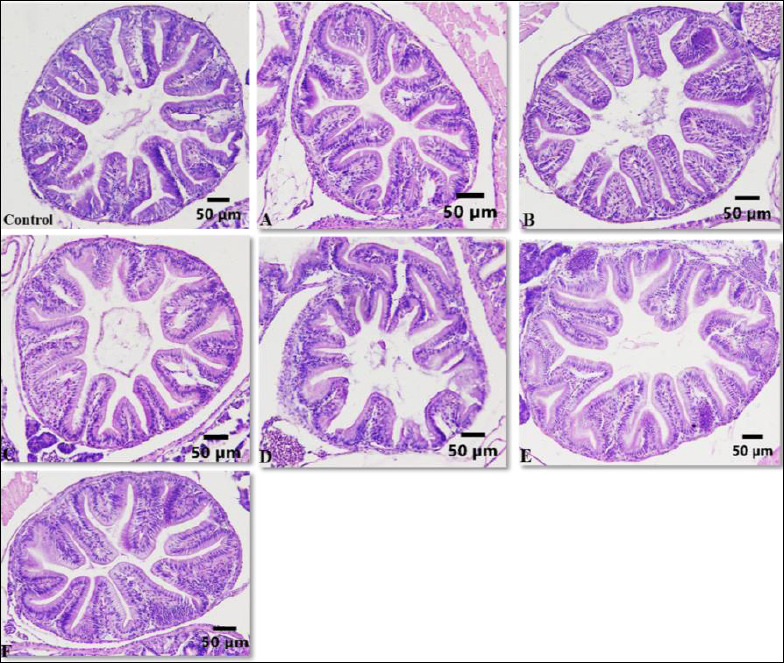
Histological cross-sections of the intestine of *Anguilla bicolor bicolor* glass eels fed *Artemia* enriched with *Porphyridium cruentum* at varying densities and enrichment durations.

**Figure 5:**
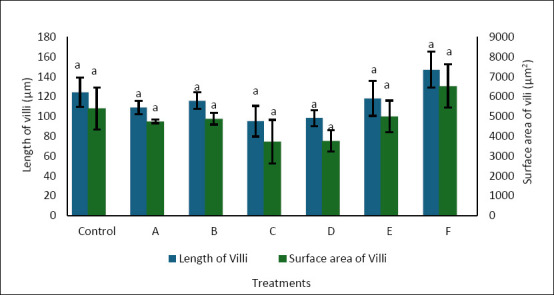
Villus length (μm) and villus surface area (μm²) in the intestine of *Anguilla bicolor bicolor* glass eels following feeding with *Artemia* enriched with *Porphyridium cruentum* under different density and enrichment time combinations.

Histological examination of the intestine at the end of the rearing period mostly showed uniform villus structure across treatments (Figure 4). No significant differences were seen between the control and enriched groups. This was supported by morphometric analysis, where villus length ranged from 95.00 to 147.06 µm and villus surface area ranged from 3,717.25 to 6,527.76 µm², with no significant differences among treatments (p > 0.05) (Figure 5). These results suggest that enrichment with *P. cruentum*, regardless of cell density or duration, did not change intestinal histology, and the basic digestive structure remained similar across treatments.

Intestinal villi are finger-like projections that enhance the surface area for absorption, thereby aiding in the uptake of nutrients and water. Villus height and surface area are frequently used as markers of nutrient absorption ability [39]. Increased villus height, fold number, and surface area generally correlate with better nutrient absorption and utilization because a larger epithelial surface is available for diffusion and active transport [40].

In the present study, although enrichment with *P. cruentum* significantly enhanced the growth performance of *A. bicolor bicolor*, no corresponding changes were observed in villus morphometry. Villus length and surface area remained relatively unchanged across treatments, indicating that dietary enrichment did not cause histological adaptation in the intestine. Therefore, the increased growth seen in treatment C cannot be attributed to an expanded intestinal structural capacity.

These results indicate that *P. cruentum* mainly contributed by increasing metabolic energy availability rather than altering intestinal structure. Red microalgae are rich in polysaccharides, proteins, and functional lipids such as PUFA, which can boost energy density and metabolic efficiency in larvae [22, 34]. Therefore, the extra energy supplied through enriched *Artemia* was more likely used for somatic growth and metabolic activities without changing villus morphology. In summary, *P. cruentum* enrichment seems to support the growth of *A. bicolor bicolor* by improving nutrient and bioactive compound availability while keeping the intestinal structure stable.

### Water quality parameters

Water quality remained fairly constant across treatments during the rearing of *A. bicolor bicolor* (Table 3). DO ranged from 4.60 to 6.50 mg/L, temperature from 26.80°C to 28.00°C, pH from 7.26 to 8.55, and total ammonia nitrogen (TAN) from 0.001 to 0.448 mg/L. These values stay within acceptable ranges for the growth and development of *A. bicolor bicolor* and suggest that no significant environmental fluctuations took place during the culture period. This stability also indicates that using *Artemia* enriched with *P. cruentum* did not negatively impact water quality.

**Table 3:** Ranges of water quality parameters (dissolved oxygen [DO], temperature, pH, and total ammonia nitrogen [TAN]) recorded during the 60-day rearing period of *Anguilla bicolor bicolor* glass eels under different *Artemia* enrichment treatments.

Treatment	DO (mg/L)	Temperature (°C)	pH	TAN (mg/L)
Control	4.6–6.4	26.8–28.0	7.26–8.18	0.275–0.428
A	4.6–6.3	26.8–27.8	7.91–8.48	0.001–0.133
B	4.6–6.5	26.8–28.0	7.92–8.45	0.120–0.164
C	4.6–6.5	26.8–27.9	7.94–8.48	0.276–0.448
D	4.6–6.5	26.8–27.9	7.93–8.49	0.076–0.394
E	4.6–6.4	26.8–27.8	7.93–8.55	0.040–0.253
F	4.7–6.4	26.9–27.9	7.93–8.40	0.273–0.403

High-quality and digestible live feed like *Artemia* is known to reduce uneaten feed residues and nitrogenous waste, thereby decreasing the risk of water pollution [41]. In this study, enrichment with *P. cruentum* not only enhanced the nutritional profile of *Artemia*, including water-soluble proteins, PUFA, and antioxidants, but also seemed to support environmental stability. Red microalgae are recognized for their ability to absorb nitrogen, which may indirectly help keep TAN within safe limits [42]. Additionally, several compounds in *P. cruentum* have antioxidant, anti-inflammatory, and immunomodulatory properties that could reduce stress and assist in maintaining physiological homeostasis in cultured fish [43, 44].

This observation aligns with previous reports suggesting that supplementing aquafeeds with microalgal products can enhance not only nutritional quality but also fish health and water quality stability [45, 46]. Therefore, maintaining suitable water quality in this study probably reflects a positive interaction between the improved nutritional performance of enriched *Artemia* and the ecological contribution of *P. cruentum* in the rearing system.

### Bacterial dynamics in the rearing medium

**Figure 6:**
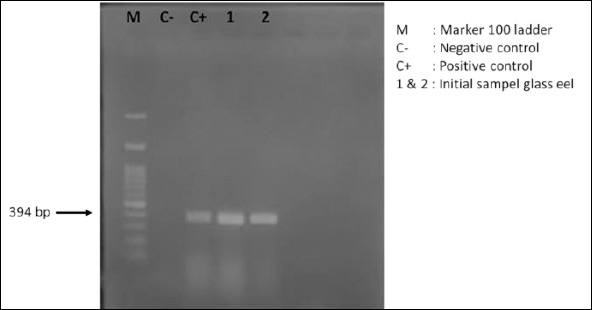
Agarose gel electrophoresis profile showing DNA amplicons of *Anguillid herpesvirus 1* in *Anguilla bicolor bicolor* glass eels prior to their use as experimental test organisms.

Although water quality stayed within acceptable limits, microbiological conditions also significantly influenced the culture environment. Total plate count (TPC) analysis revealed that bacterial counts were higher in all enriched treatments compared to the control (0.84 × 10^5^ CFU/mL), with the highest count found in treatment D (2.00 × 10^5^ CFU/mL). The other enriched groups ranged from 1.00 × 10^5^ to 1.80 × 10^5^ CFU/mL (Figure 6). Despite this increase, all counts remained within levels typically regarded as safe for cultured fish.

The higher bacterial counts in treatments receiving *P. cruentum*-enriched *Artemia* may be attributed to two factors. First, enriched *Artemia* might have carried additional biomolecules, such as water-soluble proteins, sulfated polysaccharides, and lipophilic pigments, which can serve as substrates for heterotrophic bacteria [36, 38]. Second, red microalgae like *P. cruentum* are known to produce EPS [35], which may act as carbon sources for microbial communities while also providing immunostimulatory effects in fish.

Moderate bacterial abundance may not necessarily be harmful. Non-pathogenic bacterial communities at low to moderate levels can prevent colonization by pathogenic bacteria through competition for nutrients and space, while also producing antagonistic bioactive metabolites [47]. Therefore, the increase in TPC observed in enriched treatments should not be seen only as a negative result but may instead reflect a more active and potentially beneficial microbial environment.

Importantly, the increased bacterial load did not harm growth performance or survival. This indicates that the beneficial nutritional and bioactive properties of *P. cruentum* outweighed any potential risks linked to higher bacterial levels. Therefore, enrichment with *P. cruentum* not only enhanced the nutritional quality of *Artemia* but may also have affected microbial interactions in the culture system in a way that is compatible with glass eel rearing.

### Detection of AngHV-1 infection

Molecular screening conducted before the experiment confirmed that the *A. bicolor bicolor* population used in this study was already infected with AngHV-1 (Figure 6). This finding aligns with previous reports showing that wild-caught glass eels commonly harbor viral infections, which pose a major challenge for aquaculture [48, 49]. Semi-quantitative assessment based on DNA band intensity indicated that infection levels were already high at the start of rearing.

**Figure 7:**
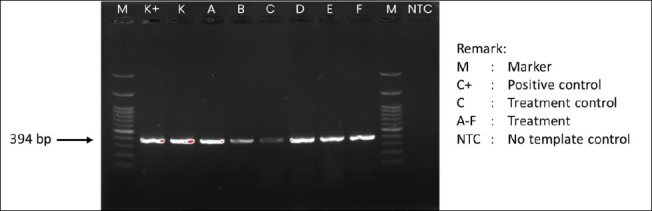
Agarose gel electrophoresis profile showing DNA amplicons of *Anguillid herpesvirus 1* in *Anguilla bicolor bicolor* glass eels after 60 days of rearing under the experimental treatments.

After 60 days, polymerase chain reaction analysis showed that AngHV-1 genomic DNA was still detectable in all experimental groups, although band intensity varied among treatments (Figure 7). The control and treatments A, B, D, E, and F exhibited relatively thick bands, indicating severe infection. Treatment B had a moderate band intensity, while treatment C showed only a faint band, consistent with mild infection. These findings suggest that dietary supplementation through *Artemia* enriched with *P. cruentum* may lessen the severity of AngHV-1 infection, although it does not completely eliminate the virus.

This pattern can be understood within the framework of herpesvirus biology. As part of the Herpesviridae family, AngHV-1 establishes lifelong latency, with the viral genome remaining in host cell nuclei as episomes and reactivating under stress or immunosuppression [50]. Therefore, although viral DNA was detectable in all treatments, the fainter bands in treatments C and B indicate lower viral activity and suggest the virus may be in a more latent state. This suggests that improved physiological and immunological health in the enriched groups may have suppressed viral replication.

These results also align with previous reports indicating that AngHV-1 is widespread among various *Anguilla* species, including *A. anguilla*, *A. japonica*, *A. rostrata*, *A. bicolor bicolor*, and *A. marmorata* [48, 49, 51–54]. Since artificial propagation of glass eels is not yet available [55, 56], eel aquaculture still relies on wild-caught seed that may already carry infections before entering culture systems. Given these circumstances, completely preventing infection is unrealistic, and management should focus on minimizing viral impact rather than stopping exposure.

Since herpesviruses are obligate intracellular pathogens, direct chemotherapeutic control is highly limited [48]. Therefore, managing AngHV-1 must depend on non-drug approaches. These can include functional feeds and immunostimulants, vaccination, and environmental adjustments such as temperature control to inhibit viral replication [52]. Elevated temperatures have also been reported to boost antiviral responses by increasing the expression of immune-related genes, including interferon-γ, interleukin-1β, and Mx1 [20]. While these methods may not eradicate the virus, they can decrease viral replication and reduce the risk of transmission.

In the present study, the relationship among survival (Figure 1), growth performance (Figures 2 and 3), and AngHV-1 band intensity (Figures 7 and 8) indicates that enrichment with *P. cruentum* supports the physiological condition of *A. bicolor bicolor* in a way that promotes improved performance while reducing apparent viral severity. Collectively, these results suggest that nutritional management through *P. cruentum* bioencapsulation plays a key role in mitigating infection severity and maintaining the performance of *A. bicolor bicolor* glass eels during culture, even though AngHV-1 remains detectable in all groups.

### Study limitations

Several limitations should be considered when interpreting the present findings. First, AngHV-1 load was assessed using conventional polymerase chain reaction and semi-quantitative visual evaluation of band intensity, without employing an internal control gene or quantitative PCR-based quantification. Therefore, the differences in AngHV-1 levels among treatments should be regarded as approximate and require confirmation using more sensitive, quantitative molecular methods. Second, intestinal histological assessment was performed on a limited number of fish and villi per treatment, which may have reduced the ability to detect subtle morphometric differences and could explain the lack of significant variation in villus structure despite the notable improvements in survival and growth.

Third, only total heterotrophic bacterial counts were measured, without distinguishing beneficial bacteria from potentially pathogenic taxa. Additionally, water quality was evaluated at specific sampling points rather than continuously, which limited the temporal resolution of microbial and environmental fluctuations during the culture period. Finally, the experiment was conducted at a pilot semi-indoor scale for 60 days with a single cohort of glass eels from one geographic source. Therefore, caution is necessary when extrapolating these findings to other eel stocks, hatchery systems, or long-term production conditions.

### Future perspectives

Future research should quantify AngHV-1 dynamics using quantitative PCR and include immune-related endpoints, such as the expression of antiviral and pro-inflammatory genes. It should also incorporate controlled challenge studies to better clarify how *P. cruentum*-enriched *Artemia* affects viral replication and host defense. A more comprehensive histological and functional assessment of the digestive tract, including digestive enzyme activity and gut barrier markers, would also help differentiate the relative roles of structural and metabolic factors in improving growth performance.

Additionally, more detailed profiling of crude lipid, fatty acids, and pigments in enriched *Artemia*, along with characterization of related microbial communities, would offer a more thorough understanding of how *P. cruentum* alters the nutritional and microbial environment of glass eels. Finally, validation in commercial hatchery settings, including economic analysis and integration with complementary management strategies such as temperature control or vaccination, is necessary to assess the practicality of *P. cruentum*-enriched *Artemia* as a non-pharmaceutical approach for managing AngHV-1 in tropical eel aquaculture.

## CONCLUSION

This study showed that enriching *Artemia* with *P. cruentum* significantly enhances the nutritional quality of live feed and improves the survival and growth of *A. bicolor bicolor* glass eels under AngHV-1 endemic conditions. Among the tested treatments, enrichment at 6 × 10^5^ cells/mL for 6 h produced the best results, achieving the highest survival rate (60.74% ± 3.9%) and SGR (1.11% ± 0.1%/day), along with a notable boost in *Artemia*’s nutritional profile, including crude protein, crude lipid, and gross energy content. Despite these notable improvements in survival and growth, intestinal villus morphology remained similar across treatments, suggesting that performance gains were mainly due to enhanced nutrient availability and bioactive compound delivery rather than structural changes in the digestive tract. Additionally, water quality parameters and bacterial levels stayed within acceptable ranges for eel culture, confirming that *P. cruentum* enrichment did not negatively impact the rearing environment.

From a practical standpoint, these findings emphasize the potential of *P. cruentum*-enriched *Artemia* as an effective live feed strategy to improve early rearing success of *A. bicolor bicolor* in hatchery systems that depend on wild-caught glass eels. The decrease in AngHV-1 DNA band intensity seen in the optimal treatment indicates that better nutritional status and the presence of bioactive compounds from *P. cruentum* may help increase host resilience and partially suppress viral activity. Since there are no current direct antiviral treatments for AngHV-1, using enriched live feeds for nutritional management offers a practical, non-pharmacological way to reduce disease impacts in eel aquaculture.

A major strength of this study is its comprehensive evaluation of nutritional, physiological, microbiological, and virological factors within a single experimental setup that mimics hatchery conditions. By analyzing feed composition, growth performance, intestinal structure, water quality, bacterial behavior, and AngHV-1 infection status simultaneously, the study offers a thorough understanding of how functional live feed enrichment affects both fish performance and the culture environment.

Overall, the results show that enriching *Artemia* with *P. cruentum* at 6 × 10^5^ cells/mL for 6 h is an effective nutritional strategy for boosting survival and growth while enhancing host resilience against AngHV-1 in *A. bicolor bicolor* glass eels. These findings provide a scientific basis for integrating microalgal enrichment into eel hatchery management and support the development of more sustainable and biosecure tropical eel aquaculture systems.

## DATA AVAILABILITY

All data generated or analyzed during this study are included in this manuscript. Additional supporting data are available from the corresponding author upon reasonable request.

## AUTHORS’ CONTRIBUTIONS

AS: Conceptualization, data curation, formal analysis, investigation, methodology, and writing of the original draft. LHS: Data curation, formal analysis, project administration, visualization, writing of the original draft, and writing–review and editing. EKMK: Conceptualization, investigation, methodology, and data curation. AN: Investigation, formal analysis, methodology, resources, and writing of the original draft. EBK: Data curation, resources, formal analysis, and writing–review. TT: Investigation, formal analysis, methodology, resources, and writing of the original draft. YRW: Supervision, validation, writing, and resources. RS: Formal analysis, validation, writing of the original draft, and resources. SM: Project administration, methodology, resources, and writing–review and editing. LR: Formal analysis, validation, writing of the original draft, and resources. THP: Data curation, formal analysis, methodology, and supervision. BP: Resources, formal analysis, validation, writing–review and editing, and supervision. TB: Conceptualization, investigation, methodology, data curation, and supervision. MM: Validation, resources, and writing–review and editing. ES: Conceptualization, investigation, methodology, and data curation. HH: Validation, writing–review and editing, and supervision. All authors have read and approved the final manuscript.
